# Drug-Eluting Intraocular Lenses

**DOI:** 10.3390/ma4111927

**Published:** 2011-11-01

**Authors:** Clara González-Chomón, Angel Concheiro, Carmen Alvarez-Lorenzo

**Affiliations:** Departamento de Farmacia y Tecnología Farmacéutica, Facultad de Farmacia, Universidad de Santiago de Compostela, 15782-Santiago de Compostela, Spain; E-Mails: clara.gonzalez@usc.es (C.G.-C.); angel.concheiro@usc.es (A.C.)

**Keywords:** intraocular lens, drug delivery, combination product, hydrogel, drug soaking, supercritical fluids, posterior capsule opacification

## Abstract

Notable advances in materials science and in surgical techniques make the management of cataract by replacement of the opaque crystalline with an intraocular lens (IOL), one of the most cost-effective interventions in current healthcare. The usefulness and safety of IOLs can be enhanced if they are endowed with the ability to load and to sustain drug release in the implantation site. Drug-eluting IOLs can prevent infections and untoward reactions of eye tissues (which lead to opacification) and also can act as drug depots for treatment of several other ocular pathologies. Such a myriad of therapeutic possibilities has prompted the design of drug-IOL combination products. Several approaches are under study, namely combination of the IOL with an insert in a single device, soaking in drug solutions, impregnation using supercritical fluids, coating with drug/polymer layers, and covalent grafting of the drug. The advantages/limitations of each technique are discussed in the present review on selected examples. Although more *in vivo* data are required, the information already available proves the interest of some approaches in ocular therapeutics.

## 1. Intraocular Lenses (IOLs) 

Vision loss due to cataracts has represented an important concern along the human history and is far from diminishing; its incidence is rising as life expectancy and prominence of adverse agents (UV irradiation, smoke) or conditions (diabetes, malnutrition) increase [1,2,3,4]. The cataract term refers to the opacification of the crystalline lens or the anterior or posterior part of the lens capsule. Osmotic changes, protein aggregation and slow down of the metabolic processes alter the proper transmission of light through the lens [5]. The first documented attempts to replace the natural opacified lens for a glass substitute date of XVIII century, but it was not until the mid-20th century that intraocular implantation of compatible materials was possible [6]. The fortuitous finding that small pieces of the transparent plastic components (poly(methylmethacrylate), PMMA) of the Second World War fighter planes did not cause damage in eyes of the pilots led Dr. H. Ridley to think about how to apply them to replace the natural lens during cataract surgery. Ridley, together with an optical scientist, J. Pike, asked the Plastics Division of the Imperial Chemical Industries for preparing highly pure (clinical grade) PMMA, that was tested for first time in 1949 [7]. Although Ridley tried to maintain this innovation under secrecy for some years to collect data about the performance of the material *in vivo*, soon his colleagues realized what was happening. The initial adverse comments about the insertion of PMMA gradually moved to general acceptance of this practice, which can be considered as one of the first examples of replacement of a damaged tissue by an artificial device able to almost fulfill the natural function [7]. Notable advances in the materials used for intraocular lenses (IOLs) and in surgical techniques, have taken place in the last decades [8,9]. These improvements make the extraction of the natural opaque crystalline and the replacement by an IOL one of the most cost-effective interventions in current healthcare [10]. The field of IOLs now extends to refractive surgery procedures useful for permanent and accurate correction of common vision errors such as presbyopia, myopia or hyperopia [9], which may involve clear lens extraction (CLE), namely the removal of the noncataractous natural lens and replacement with an IOL of adequate corrective power; or just the insertion of a corrective lens (phakic implantable lens) in front of the eye’s natural lens [11].

Since IOLs are designed to remain inside the eye for a prolonged time, they have to accomplish several features, namely optical properties similar to those of the natural lens (density, refractive index, transmittance), biocompatibility and long term stability [9]. Furthermore, their morphology and mechanical properties have to match to the minimally invasive insertion practices. IOLs can be classified into several groups as a function of the constitutive polymer [1,6,9], as follows: 

(i) PMMA provides biocompatible IOLs with a high refractive index but, since it is a rigid material, its insertion requires large incisions and is associated to endothelial damage and post-operative adhesion of inflammatory cells [6]. These drawbacks have prompted the search for flexible/foldable materials, described below. 

(ii) Synthetic polysiloxane (silicone), such as poly(dimethylsiloxane) and poly(diphenylsiloxane), enables the production of flexible and soft IOLs with low affinity for water. They have a low refractive index and are not recommended in patients in which silicone oil is used as vitreous substitute [9]. 

(iii) Acrylic polymers provide IOLs that combine the advantages of both PMMA and silicone, *i.e*., suitable refractive index and tunable foldability. Thin IOLs prepared with acrylic components require minor incisions for implantation and minimize post-surgery complications [6]. Hydrophobic acrylic IOLs made of esters of poly(meth)acrylic acid absorb less than 1% water and remain foldable even when they are totally dry. Recent research is being focused on shape-memory acrylic materials that can be inserted predeformed and fixed to quite small size and that recover the adequate shape because of the responsiveness to the body temperature [12,13]. Hydrophilic acrylic IOLs, mainly constituted by 2-hydroxyethyl methacrylate (HEMA) and 6-hydroxyhexyl methacrylate (HEXMA), are flexible when wet (18–38% water) but become rigid and unfoldable when dried [8,9]. Phakic IOLs combine HEMA with collagen (Collamer lenses, [11]). 

Postoperative ocular inflammation and posterior capsule opacification (PCO) are the main concerns of the IOL implantation. Epithelial cell adhesion, growth and proliferation on the posterior side of the lens capsule and the IOL may cause loss of vision in months/years after IOL implantation (secondary cataract). Retained cortical fibers and bladder, fibrocyte-like and myoepithelial cells can also contribute to the PCO [6]. The growth of the epithelial cell depends on the IOL material and the monocyte/macrophages reaction. IOLs having low contact angle are more biocompatible, mainly because the hydrophilic surfaces hinder the adsorption of proteins (fibronectin, vitronectin, laminin, hyaluronan, collagen) that precedes to the extracellular matrix formation and serves as receptor of the cells [9]. Nevertheless, even the most biocompatible IOL material induces certain grade of inflammatory cell adhesion. The high incidence of the PCO and the relatively high cost of capsulotomy by Nd:YAG laser makes the treatment of PCO quite expensive [6]. Therefore, prevention of PCO has a great medical and economical relevance. Several prophylactic strategies are focused on physical features of the materials, such as design of IOLs with sharp edge, less roughness, low water contact angle, or coated with protein/cell repellent substances [6,14].

Intraocular administration of antibodies against epithelial cells or drugs that destroy the epithelial cells at the moment of insertion has the inconvenience that it decays within a time period too short to prevent PCO. To overcome these limitations, several approaches to develop IOL/drug combination products are under evaluation [15]. These combination products can not only prevent PCO but may also help in the treatment of concurrent ocular pathologies (e.g., severe uveitis, age related macular degeneration or proliferative diabetic retinopathy) by sustaining the release in the posterior chamber of steroidal and nonsteroidal anti-inflammatory drugs (fluocinolone acetonide, indomethacin, diclofenac sodium, flurbiprofen), antineoplasic agents (daunorubicin, mitomycin C, 5-fluorouracil), and other active substances such as colchicine, EDTA, rapamycin or the fibroblast growth factor 2-saporin [16,17,18,19]. Although less frequent, infections associated to the cataract surgery and also to the phakic lens implantation may become a relevant complication, with an incidence of up to 0.3% [20,21]. Treatment of endophthalmitis usually requires intraocular injections and, if failure occurs, the removal of the infected IOL. Therefore, prophylactic strategies such as incorporation of antimicrobial polymers [4] and/or sustained release of antimicrobial agents from the IOLs themselves are gaining increasing attention [22,23,24,25]. The information currently available about IOL/drug combination products is analyzed in the next section.

## 2. Drug Loading and Elution from IOLs 

Several approaches are under study to endow IOLs with the ability to host drugs and to sustain its release in order to avoid PCO and to treat other various ocular diseases. The drug-IOL combination products [15] may be the result of the loading of commercially available IOLs with specific drugs or of the design of IOLs with particular composition and microstructure in order to improve the drug loading and to achieve an efficient control of the release.

### 2.1. Pendant Solvent-Cast Drug Inserts

**Figure 1:**
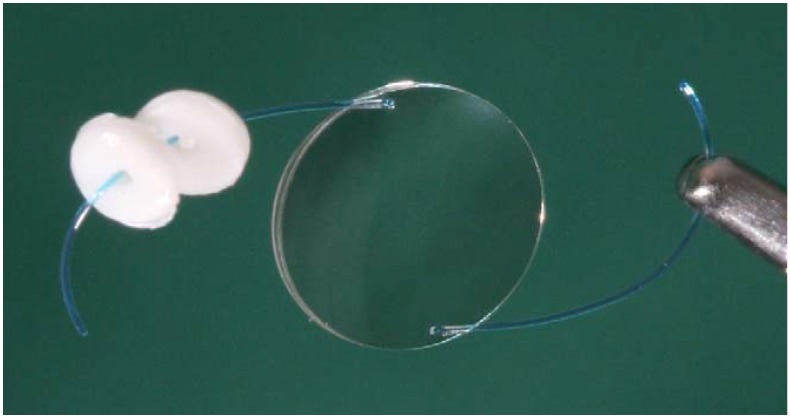
Picture of the intraocular lens (IOL) combined with two drug delivery systems. Reproduced from reference [27] with permission of Elsevier.

Combination of an IOL with one or more drug-loaded inserts in a joining device (Figure 1) is perhaps the most direct approach to achieve intraocular release in the environment surrounding the IOL [26,27]. The current knowledge about design and synthesis of biodegradable inserts leads to suitable intraocular drug depots that efficiently regulate the release rate [28,29]. It has been previously shown that simultaneous insertion of single IOLs and corticosteroids implants during cataract surgery provide good clinical results in eyes affected by severe conditions, such as uveitis [30]. The physical link of the IOL and the insert (Figure 1) has the advantage that the insert is not free to move to other eye regions compromising visual acuity, and that the composition of the IOL does not have to be modified since they are separately prepared [31]. The major difficulty is to effectively link the insert to the IOL. Although research on this approach is still incipient, tests in animal models evidenced that IOLs coupled to two inserts of poly(d,l-lactide-co-glycolide), PLGA, loaded with triamcinolone acetonide enabled a long-term reduction of the postoperative ocular inflammation [27].

### 2.2. Drug Soaked IOLs

IOLs themselves can be loaded with drugs through immersion in concentrated aqueous or hydroalcoholic solutions (pre-soaking), as tested with soft contact lenses [22,32,33,34]. This relatively simple method is only adequate for drugs that possess affinity with the polymer network and that, consequently, can be uptaken and retained in the matrix. In addition to the match of the chemical groups of the drug and the IOL, several other factors (e.g., drug concentration in the loading medium and loading time) determine both the yield and the rate of *in vitro* drug loading and *in vivo* drug release.

**Figure 2:**
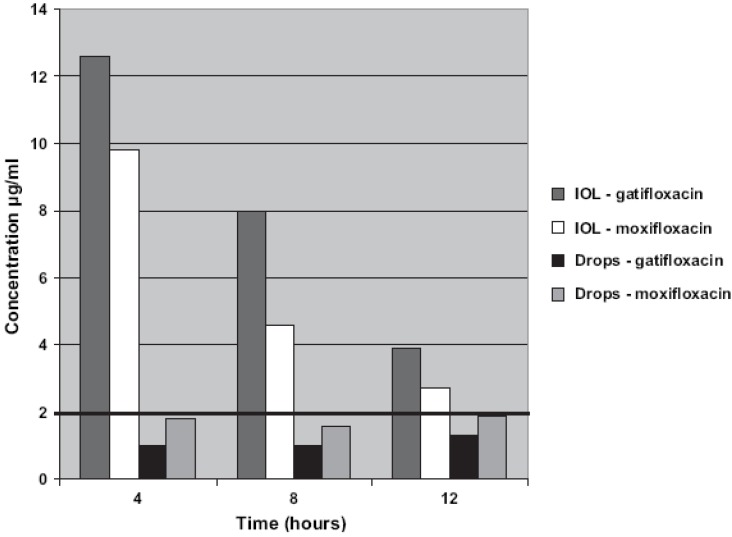
Gatifloxacin and moxifloxacin concentration in rabbits aqueous humor after implantation of drug-soaked IOLs or topical postoperative administration of drops to eyes that underwent non-soaked IOL implantation. In both groups, there was no statistically significant difference between the concentrations of the two antibiotics. Reproduced from reference [35] with permission of Elsevier.

Hydrophilic acrylic commercial lenses (C-flex, Rayner Intraocular Lenses, Ltd., East Sussex, UK) have shown affinity for fourth-generation fluoroquinolones when soaked for 24 h in moxifloxacin (5 mg/mL) or gatifloxacin (3 mg/mL) [35]. Implantation in rabbit eyes indicated that the IOLs were able to progressively release the drugs to the aqueous humor, providing gatifloxacin levels higher than those of moxifloxacin. The drug-soaked IOLs did not cause adverse reactions in the eye. Gatifloxacin concentrations at 4 and 12 h were 12.61 and 3.87 µg/mL, respectively, while moxifloxacin concentrations were 9.78 and 2.66 µg/mL (Figure 2). These concentration values are above the minimum inhibitory concentration (MIC_90_) of common infection agents, suggesting that the drug-loaded IOLs can efficiently prevent bacterial endophthalmitis. Such a good performance clearly contrasts with the low drug levels attained when the drugs are preoperative and postoperative applied as eye-drops during non-soaked IOLs implantation (Figure 2). A later study confirmed that another hydrophilic IOL, namely STAAR Collamer^®^ (composed by collagen and poly-HEMA copolymers) can also be loaded by soaking in moxifloxacin, gatifloxacin, linezolid and cefuroxime solutions for 1 h. When implanted in rabbit eyes, the drug-loaded IOLs provided therapeutic concentrations in aqueous and vitreous humor, remaining above the MIC for at least 6 h, and even for 24 h in the case of gatifloxacin [36].

The influence of the IOL composition and the time spent soaking has been analyzed in a comparative study that involved hydrophobic (AcrySof^TM^ SA60, Alcon Inc., Fort Worth, TX, USA) and hydrophilic (Afinity^TM^ CQ2015, STAAR Surgical Company, Monrovia, CA, USA) acrylic lenses. The lenses were soaked in 1 mL of 0.5% moxifloxacin ophthalmic solution for 1 or 10 minutes, and then the release was monitored by placing in 10 mL of balanced salt solution for 30 min. The hydrophobic IOLs presoaked for 1 or 10 min released 2.4 ± 2.1 µg and 3.4 ± 3.9 µg of moxifloxacin, respectively. Under the same conditions the hydrophilic IOLs released 2.8 ± 1.0 µg and 7.2 ± 4.3 µg [24]. No influence of the IOL nature and the soaking times was observed. In any case, the mean antimicrobial drug concentration achieved in the release medium was well above the MIC_90_ of *Staphylococcus epidermidis*, *S. aureus* or *Haemophilus influenzae* [35]. These data agree well with the inhibition zones observed in *in vitro* microbiological tests carried out with AcrySof^TM^ SA60 IOLs soaked for 1 or 60 min in moxifloxacin or gatifloxacin solutions; no significant improvement was observed when the time of soaking increased [22]. Thus, 1 min soaking may be sufficient for obtaining IOLs able to effectively prevent infectious complications of cataract surgery and, if an adequate soaking protocol is followed in the operation room, the time of soaking is sufficiently short to not cause a relevant extent of the cataract surgery.

The ability of hydrophilic acrylic IOLs (*i.e*., H60M; Bausch and Lomb, Rochester, NY, USA) to load other drugs was tested by immersion in tranilast (1 mg/mL), diclofenac sodium (0.2 mg/mL), mitomicin C (0.2 mg/mL), colchicine (12.5 mg/mL) and 5-fluorouracile (10 mg/ml) sterile solutions at 37 °C for 3 h. Inhibition of the adhesion and growth of lens epithelial cells on the IOL was observed for those loaded with tranilast, diclofenac sodium and 5-fluorouracile. Therefore, IOLs loaded with these drugs can prevent PCO [37].

The suitability of silicone IOLs to act as drug carriers has also been evaluated by presoaking in a wide range of active substances. CeeOn^®^ (AMO, Santa Clara, CA, USA) IOLs were incubated in dexamethasone (1 mg/mL) in ethanol:water 50:50 for 40 min with the purpose of preventing the post-surgery inflammation [34]. After washing with sterile water and air-dried, drug-loaded IOLs were placed in rabbit eyes and the evolution of inflammation markers was monitored and compared to the values obtained in a parallel study with the non-soaked IOLs. Concentrations of prostaglandin E2 (PGE2), proteins and white blood cells (WBCs) in aqueous humor were clearly lower when the drug-loaded IOLs were inserted. Furthermore, no PCO was observed in the 27 days of study [34]. Soaking of silicone IOLs with inhibitors of matrix metalloproteinases (MMPs) has been assayed to prevent the PCO [16]. Poly(dimethyl)siloxane disks were immersed in GM6001 and MMP 2/9 Inhibitor II ethanol solutions for 4 days. Alternatively, the MMP inhibitors were incorporated to the silicone rubber as dimethylformamide solutions before the curing of the rubber at 37 °C for 48 h. *In vitro* release tests carried out in phosphate buffer saline pH 7.4 at 37 °C evidenced that disks loaded by soaking released all drug in few hours. By contrasts, the disks to which the drug was incorporated during curing showed a burst of 10–25% in the first 24 h followed by a sustained, constant-rate delivery for the following 2–4 months.

The *in vivo* data currently available clearly indicates that the soaking in drug solutions may be a suitable way for preparing medicated IOLs in a cost-effective way and with a therapeutic performance similar or even better than other approaches, such as inserts or intracameral injections. Nevertheless, the soaking approach seems to be more adequate for hydrophilic IOLs than for hydrophobic acrylic or silicone rubber IOLs, which can be attributed to the role of the aqueous phase of the IOL network in drug hosting and also in facilitating drug diffusion into the network. Inefficient loading has also the drawback of the waste of drug in the soaking solution, with the associated economical and environmental concerns. Thus, several other approaches to prepare drug-IOL combination products are under evaluation.

### 2.3. Supercritical Fluids Impregnation

**Figure 3:**
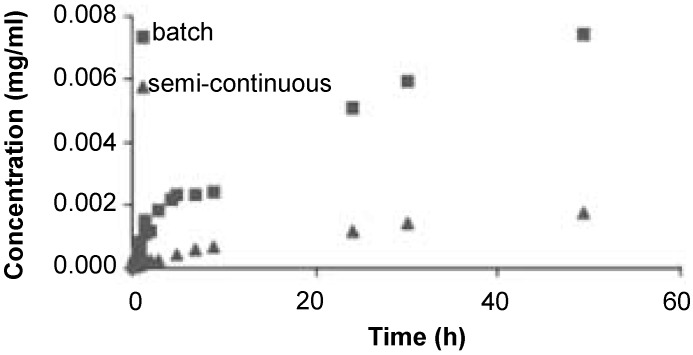
Release profiles from flurbiprofen-impregnated IOLs that were processed according to a batch or a semi-continuous way. The percentages of impregnation, defined as the relative quantity of drug in an impregnated sample, were 0.82% and 0.22% respectively. Reproduced from reference [19] with permission of Elsevier.

Supercritical fluid-based technologies are attracting growing attention as a way to force the entrance of drug molecules in biomaterials networks without using organic solvents and according to the green chemistry principles [19,38]. Supercritical CO_2_ (scCO_2_) is in general a better solvent for drugs than water and also a more efficient plasticizing of hydrophobic polymer networks. As a consequence, drug diffusion into the polymer matrix becomes favored and drug impregnation using scCO_2_ may result in a higher drug loading yield than conventional presoaking in aqueous medium, on a reproducible and commercially suitable scale [38,39]. Although the application of this technology to the IOLs is still incipient, promising results have already been reported. Hydrophobic acrylic lenses of PMMA copolymerized with 2-ethylhexylacrylate (EHA) were loaded with remarkable amounts of flurbiprofen by placing an amount of drug at the bottom of the impregnation cell, which was easily solubilized by scCO_2_ [19]. Experiments were carried out according to a batch mode (*i.e*., drug and IOL together in the impregnation vessel) or in a semi-continuous way (*i.e*., drug in the saturator placed before the impregnation vessel). Interestingly, a decrease in the impregnation was observed as the pressure applied increased. This finding was attributed to the fact that the increase in pressure enhances the solubility of the drug in scCO_2_ and such an increase in drug-solvent affinity decreases the tendency of the drug to adsorb on the polymer. Those IOLs loaded under the most efficient conditions were able to sustain the release of flurbiprofen for more than three months [19] (Figure 3).

In a recent paper, scCO_2_ was applied to impregnate PMMA IOLs with cefuroxime sodium and the influence of the operating conditions such as the pressure (8 to 20 MPa), the temperature (308 and 333 K), the impregnation duration (1 to 5 h), the addition of a cosolvent (ethanol) or the depressurization rate (slow and rapid depressurization) were evaluated in detail. Risk of foaming was observed during rapid depressurization. *In vitro* drug release studies showed a burst-type profile due to the drug located at/near the surface, followed by a slower release of the drug entrapped inside the polymeric matrix which confirmed an effective impregnation [40]. 

### 2.4. Deposition of Drug/Coating Components

Coating of the IOL applying layer-by-layer deposition of oppositely charged polymers that embedded the active ingredient may be an alternative suitable to coat rigid hydrophobic IOLs with a drug delivery film, if the optical properties are not affected [41]. In particular, it has been shown that PMMA lenses activated with -NH_2_ groups at the surface can be coated with successive layers of poly(sodium 4-styrenesulfonate) containing ampicillin and poly(ethylenimine). Coatings of six layers-thickness provided a sustained release of 105 µg of ampicillin for 7 days [41].

**Figure 4:**
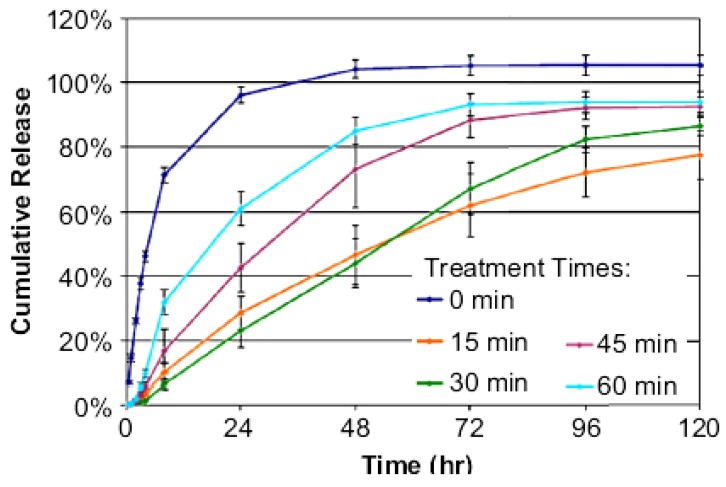
Norfloxacin release profiles from surface-modified pHEMA matrices that were treated for 15, 30, 45 or 60 min with octadecyl isocyanate. Reproduced from reference [23] with permission of Elsevier.

**Figure 5:**
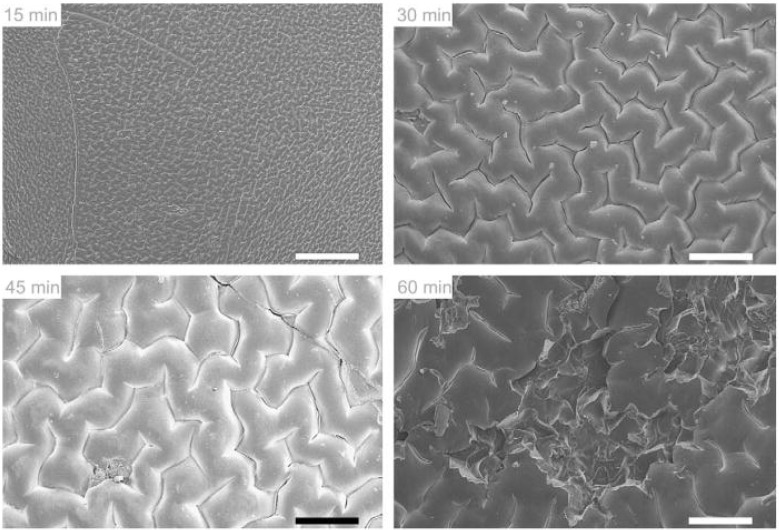
Scanning electron micrographs of octadecyl isocyanate-modified pHEMA surfaces after 15, 30, 45, and 60 min. The 15-min reaction time shows a low-amplitude reticulated pattern, while longer times show increasing crevasse size and surface damage. Scale bars are 200 μm. Reproduced from reference [23] with permission of Elsevier.

Spraying of the IOL with a coating solution may result in a faster and simplified method than the layer-by-layer deposition. It has been shown that commercial PMMA lenses sprayed with a solution of PLGA in chloroform containing an appropriate amount of rapamycin can prevent PCO [42]. Each rapamycin-PLGA-IOL contained 40 µg of drug and 10 µg of PLGA. When implanted in rabbit eyes, the drug-coated IOLs led to remarkably less accumulation of epithelial cells than those IOLs non-coated or just coated with PLGA, which evidenced multilayer cell proliferation [42]. Coating of hydrophilic acrylic (HEMA) IOLs can be achieved by short time immersion in octadecyl isocyanate solution [23]. The hydroxyl groups of pHEMA react with the isocyanate groups to form stable polyurethane bonds. The grafted polymer endowed the norfloxacin-containing IOLs with tunable release rates. Norfloxacin was added to the monomer solution before synthesizing the hydrogel. Treatment of the IOLs with octadecyl isocyanate for 15–30 min resulted in a dense coating able to delay drug release (Figure 4). Longer treatment resulted in a faster release, although still slower than from non-coated IOLs, which has been attributed to the disruption of the tightly packed methylene coating when there is an excess of n-alkyl chains and the pHEMA structure becomes sterically distorted (Figure 5). The norfloxacin release rates obtained *in vitro* suggest that the coated IOLs can erradicate *S. epidermidis* in the dynamic environment of the anterior chamber of the eye [23].

### 2.5. Chemically Grafted Drugs

Chemical binding of drug molecules to the IOLs has been investigated mainly for two purposes: (i) to endow the IOL surface with permanent therapeutic activity; or (ii) to be able to trigger drug release only under very particular conditions. Compared to the above commented approaches, the fixing of the drug to the IOL confines the therapeutic action to its closer environment without risk of collateral local effects. As an example of the first purpose, pHEMA lenses were coated with selenocystamine in order to prevent PCO [43]. To do that, an activation solution containing carbonyldiimidizole and dichloromethane was placed onto the IOL to allow the covalent linkage of selenocystamine dihydrochloride. *In vitro* cell analyses were performed with both treated and non-treated IOLs and proliferating cell nuclear antigen (PCNA), alpha-smooth muscle actin (alpha-SMA) and cleaved caspase-3 were examined by immunohistochemistry. Selenocystamine-coated IOLs did not leach toxic compounds but they effectively prevent cell growth on their surface, leading to PCO scores remarkably lower than those of non-treated IOLs under *ex vivo* conditions [43].

The cyclic exposition of the eyes to the sun light and also the feasibility of illuminating with artificial-source monochromatic light the eye structures offers interesting possibilities for obtaining triggerable drug release from IOLs. It has been shown that chemical grafting of a porphyrin photosintetizer on a hydrophilic anionic IOL, made of HEMA copolymerized with methacrylic acid, can be done by immersion in solutions of a cationic porphyrin (tetrakis(4-*N*-methylpyridyl)porphyrin) through electrostatic interactions [44]. The grafting of porphyrin notably reduced bacterial adhesion under dark conditions and more relevantly when exposed to laboratory light or to sun light (Table 1). The activity even at dark has been attributed to the fact that porphyrin enhances the hydrophilicity and provides a highly charged surface that can disrupt cell membrane of bacteria attempting to attach to the IOL. Thus, the prophyrin-grafted IOLs are capable of self-eradication of postoperative intraocular infection.

**Table 1:** Reduction in *S. epidermidis* adherence to p(HEMA-co-MAA) copolymers impregnated with a cationic porphyrin (tetrakis(4-*N*-methylpyridyl)porphyrin). Reproduced from reference [44] with permission of Elsevier.

Polymer composition % w/w p(HEMA-co-MAA)	% Reduction in bacterial adherence ± SD relative to untreated control		
	Intense light (4300 lux)	Laboratory light (1260 lux)	Dark
60/40	93.90 ± 0.59	88.76 ± 2.57	90.09 ± 4.20
70/30	94.52 ± 0.77	90.01 ± 3.59	88.21 ± 6.07
80/20	96.17 ± 0.38	92.39 ± 5.38	84.70 ± 6.78
90/10	99.02 ± 0.42	90.99 ± 6.02	91.76 ± 5.99
100/0	98.88 ± 0.92	88.42 ± 2.82	86.22 ± 6.32

**Figure 6:**
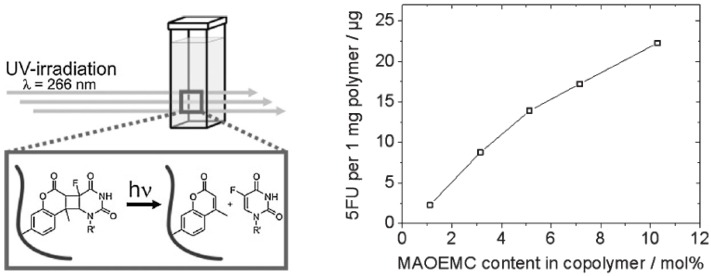
5-fluorouracil released under 266 nm irradiation from drug-7-(2’-methacryloyloxyethoxy)-4-methyl-coumarin conjugated to PMMA. Reproduced from reference [45] with permission of Elsevier.

Just recently, photoinduced release of 5-fluorouracil was achieved by immobilization of the drug into PMMA lenses through a linker group such as coumarin or a derivative, by forming a cyclobutane linkage which can be cleaved with high-energy UV irradiation [45]. A linear correlation between the degree of functionalization of the IOL and the amount of drug released was observed; the most functionalized IOL released 22.2 µg of 5-fluorouracil under UV irradiation (Figure 6) [45].

## 3. Conclusions

Endowing IOL with the ability to sustain drug release into its surroundings appears to be a very promising tool for efficient prophylaxis of secondary cataract as well as for the coadyuvant treatment of some eye pathologies. Notable advances in the drug loading techniques, novel approaches and information about the factors that condition the yield of loading and the drug release rate are expected to pave the way to the design of suitable drug-IOL combination products. In contrast with contact lenses, drug-loading treatments of IOLs are not conditioned by transparency concerns, apart from the optical portion; namely the haptics can be coated with nontransparent polymers or metallic particles, or impregnated with drugs that can tint certain regions of the IOL material. This widens the practical possibilities of using IOLs as drug delivery systems. Development of materials that combine the IOL functionality with the role of a drug delivery system and a better knowledge about the effects of drug incorporation on the IOL performance are two main pillars for a rational design of the combination products.
